# Pelvic venous disorders: I found myself within a forest dark

**DOI:** 10.1016/j.jvsv.2023.101741

**Published:** 2024-02-15

**Authors:** Sergey G. Gavrilov

**Affiliations:** Savelyev University Surgical Clinic, Pirogov Russian National Research Medical University, Moscow, Russia

With great interest I have read the review by Murali et al^1^ on iliac vein stenting for pelvic venous disorders. The presented data, unfortunately, indicate only one thing: vascular and interventional surgeons are wandering in search of optimal diagnostic and therapeutic approaches for pelvic venous disorders, especially with multifocal venous lesions. Talking about the priority of endovascular treatment, the authors refer, among other things, to one study,^2^ which, however, shows advantages of ovarian vein resection vs ovarian vein embolization in pelvic venous insufficiency (PVI) (a greater clinical efficacy and lower complication and relapse rates). A question is whether ovarian vein embolization should be performed in all PVI cases, or rather we need to focus on the patient's anthropometric parameters and anatomical features of the ovarian veins? It is not that ovarian vein embolization or ovarian vein resection is bad, but which technique is better in a particular patient? So, it is again about a personalized approach (Fig).

**Fig:**
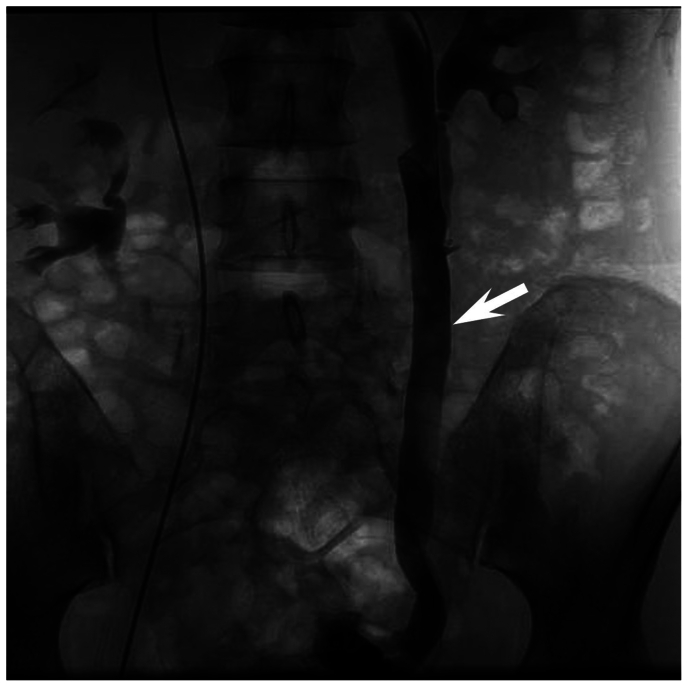
Ovarian venogram (frontal projection). The left gonadal vein diameter (arrow) is 14 mm. The patient's body mass index is 18 kg/m^2^. Would you perform an embolization?

Left common iliac vein (LCIV) compression stenosis can have at least four appearances: asymptomatic, with chronic venous disease symptoms, with PVI symptoms and pelvic varicose veins with reflux in them, or as a combination of chronic venous disease and PVI. The third type is the most difficult because it requires to find out whether PVI is caused by LCIV stenosis or primary reflux in ovarian veins. Therefore, it is of great importance to correctly interpret imaging data suggesting LCIV stenosis and to determine its hemodynamic significance, which depends, most likely, not on the grade of narrowing,^3^ but on its effect on the blood outflow through the pelvic veins and occurrence of reflux in them and collateral flow in ascending lumbar vein and the left ovarian vein.

If ovarian vein embolization eliminates PeVD symptoms in a patient with a combination of significant LCIV stenosis on venography and ovarian vein reflux, it suggests a misinterpretation of its significance for pelvic venous hemodynamics. Santoshi et al^4^ showed that ovarian vein embolization was effective in only 9.5% of 94 patients; the rest required LCIV stenting.^5^^,^^6^ Simultaneous ovarian vein embolization is probably indicated for patients with severe (>5 seconds) reflux in the left ovarian vein. The grading system for pelvic venous reflux is already used in clinical practice.^7^ Establishing clear criteria for the hemodynamic (not anatomical) significance of LCIV stenosis represents a primary task. The issue of treating patients with combined lesions of the iliac and pelvic veins is far from being solved and warrants further international randomized trials. This is the only way out of the forest dark.
